# Bacterial symbionts in oral niche use type VI secretion nanomachinery for fitness increase against pathobionts

**DOI:** 10.1016/j.isci.2024.109650

**Published:** 2024-03-29

**Authors:** Jan Oscarsson, Kai Bao, Akiko Shiratsuchi, Jonas Grossmann, Witold Wolski, Kyaw Min Aung, Mark Lindholm, Anders Johansson, Ferdousi Rahman Mowsumi, Sun Nyunt Wai, Georgios N. Belibasakis, Nagihan Bostanci

**Affiliations:** 1 Oral Microbiology, Department of Odontology, Umeå University, Umeå, Sweden; 2 Division of Oral Health and Periodontology, Department of Dental Medicine, Karolinska Institutet, Alfred Nobels Allé 8, 14104 Huddinge, Stockholm, Sweden; 3 Department of Liberal Arts and Sciences, Graduate School of Medicine, Sapporo Medical University, Sapporo, Hokkaido 060-8556, Japan; 4 Functional Genomics Center Zurich, ETH Zürich and University of Zürich, Zürich, Switzerland; 5 Swiss Institute of Bioinformatics (SIB) Quartier Sorge-Batiment Amphipole, 1015 Lausanne, Switzerland; 6 Department of Molecular Biology and the Umeå Centre for Microbial Research (UCMR), and the Laboratory for Molecular Infection Medicine Sweden (MIMS), Umeå University, 90187 Umeå, Sweden

**Keywords:** Bacteriology, Microbial flora, Microbial interactions

## Abstract

Microbial ecosystems experience spatial and nutrient restrictions leading to the coevolution of cooperation and competition among cohabiting species. To increase their fitness for survival, bacteria exploit machinery to antagonizing rival species upon close contact. As such, the bacterial type VI secretion system (T6SS) nanomachinery, typically expressed by pathobionts, can transport proteins directly into eukaryotic or prokaryotic cells, consequently killing cohabiting competitors. Here, we demonstrate for the first time that oral symbiont *Aggregatibacter aphrophilus* possesses a T6SS and can eliminate its close relative oral pathobiont *Aggregatibacter actinomycetemcomitans* using its T6SS. These findings bring nearer the anti-bacterial prospects of symbionts against cohabiting pathobionts while introducing the presence of an active T6SS in the oral cavity.

## Highlights

• Discovery that oral symbiont *A. aphrophilus* expresses type VI secretion system (T6SS)
• *A. aphrophilus* uses the T6SS to kill its pathobiont relative *A. actinomycetemcomitans*
• This *T6SS-dependent* killing was lost in mutants lacking the T6SS tube protein, Hcp

## Graphical abstract

**Figure:**
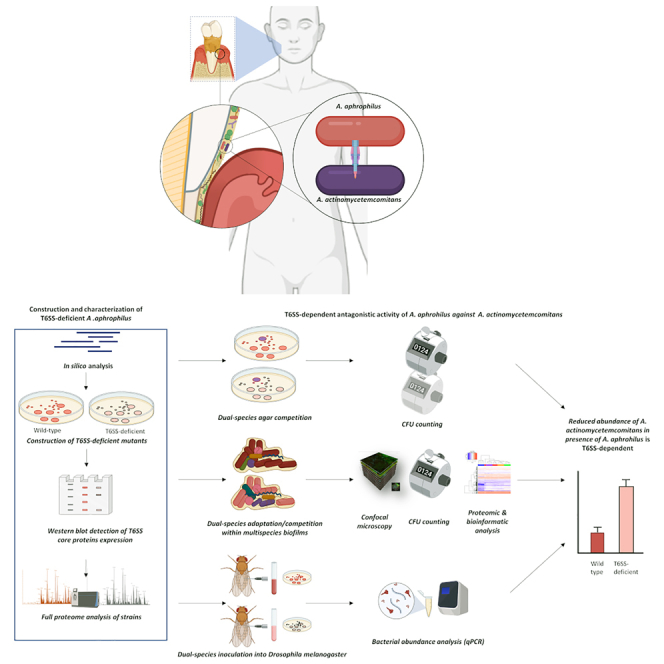

## Introduction

Bacteria utilize secretion systems that enable them to exert their influence. The type VI secretion system (T6SS) is present in approximately 25% of Gram-negative bacterial species.^1^^,^^2^^,^^3^ The T6SS enables bacteria to suppress or eliminate vulnerable prokaryotic or eukaryotic cells through the direct delivery of toxic effector proteins into the target cells, causing cell death via diverse mechanisms. Cognate immunity proteins also play an important role in protecting bacteria against their own secreted T6SS effectors.^2^ T6SS-dependent bacterial antagonism has been shown to promote the persistence of *Pseudomonas aeruginosa* in the lungs of cystic fibrosis patients,^4^ and the establishment of *Bacteroides* species, and *Salmonella* Typhimurium as members of the gut microbiota.^5^^,^^6^

Although *Aggregatibacter aphrophilus* has been reported in some cases of infectious endocarditis and brain abscesses,^7^^,^^8^ it is frequently found in the oral cavity with no association with periodontitis, which is the most prevalent oral disease and the leading cause of adult tooth loss.^9^ Its genome encodes a potential T6SS,^10^ which has not been further confirmed or studied. *A. aphrophilus* is commonly found within the oral microbial community^11^ and is considered commensal due to the lack of association with oral disease. In contrast, *Aggregatibacter actinomycetemcomitans,* closely associated with *A. aphrophilus* and sharing approximately 80% gene content,^9^ is strongly associated with infective endocarditis^12^ and aggressive forms of periodontitis in young individuals.^13^ While *A. actinomycetemcomitans* expresses unique virulence factors in the oral microbiome, such as a leukotoxin, and a cytolethal distending toxin,^14^ the presence of a T6SS has neither been confirmed nor speculated within the oral microbiome.

The natural co-habitat of *A. aphrophilus* and *A. actinomycetemcomitans* is multi-species biofilms that form on the tooth surface (dental plaque), and potentially interacting with the juxtaposed oral epithelium.^15^^,^^16^^,^^17^ Dysbiotic shifts in the microbial composition of the biofilms can permit the establishment of oral infection.^18^ Dental biofilms provide an ideal environment for studying interspecies interactions and potential roles of T6SS in these.^5^ As a putative endogenous pathogen, *A. actinomycetemcomitans* exerts ecological pressure on other biofilm co-habitant species with potentially deleterious effects on the host.^19^^,^^20^ We hypothesize that *A. aphrophilus* acts as a niche competitor by expressing a functional T6SS, which may regulate behavior and virulence of the cogenerate oral pathogen *A. actinomycetemcomitans* upon co-existence. Hence, our work aimed to validate the presence of a T6SS in *A. aphrophilus* and investigate potential antagonism toward *A. actinomycetemcomitans* in various model systems.

## Results

### Prevalence of T6SS and its gene organization in *A. aphrophilus*

**Figure 1:**
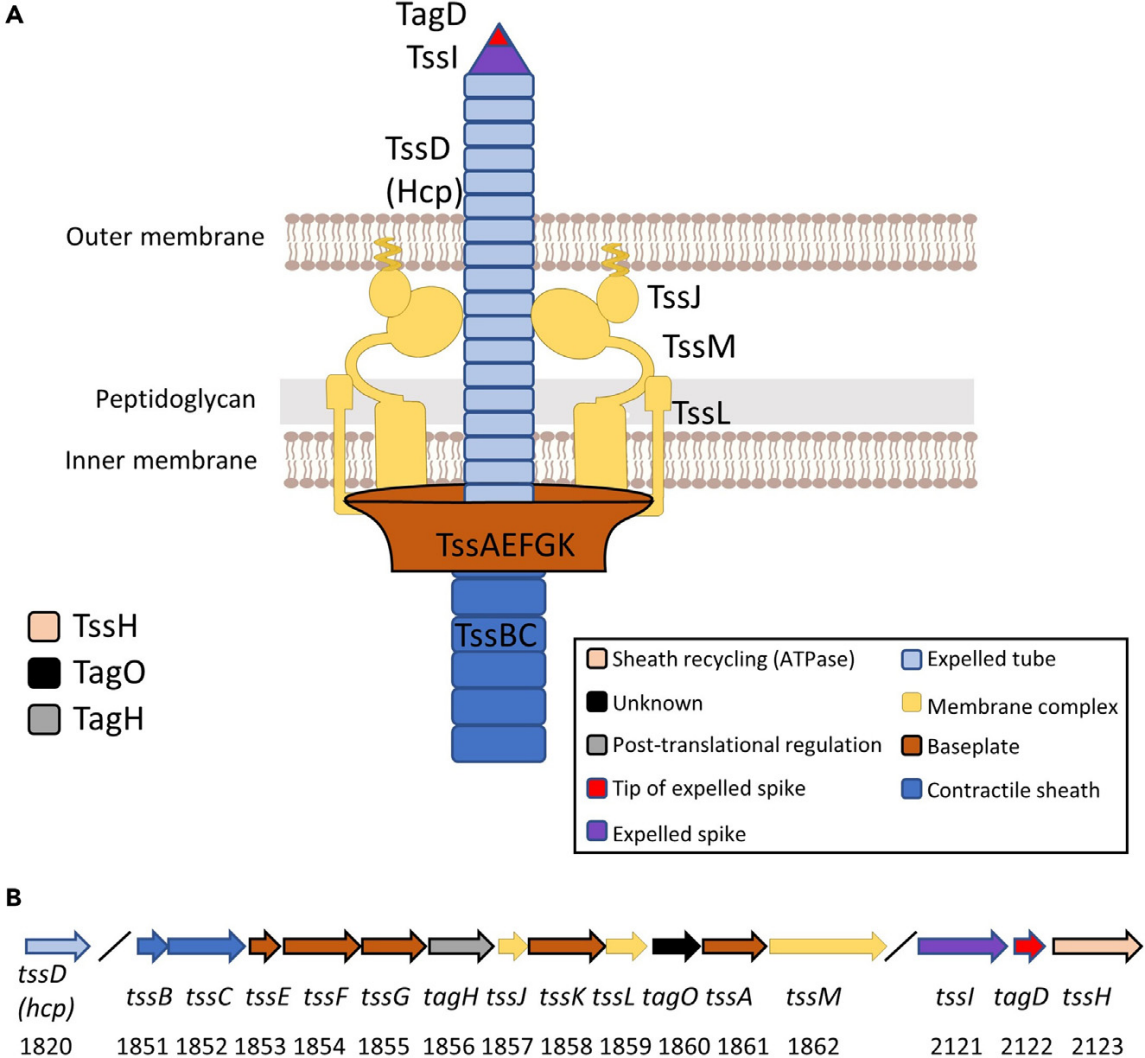
*A. aphrophilus* T6SS core components (A) Overview of predicted functions of the respective components, according to *in silico* search, and which are color-coded in both panels. More details including their alternative names and UniProtKB ID are listed in Table S2. (B) Schematic map of the T6SS-encoding main and auxiliary gene clusters, respectively, in *A. aphrophilus* reference strain NJ8700, with their respective gene numbers.^10^

To confirm and assess the prevalence of the T6SS in *A. aphrophilus*, we searched whole genome sequences (*n* = 18) of 17 strains (National Center for Biotechnology; NCBI) using BLAST. Using Tss (*tssA-M*) nomenclature for core components, and Tag (*tagA-P*) for accessory proteins,^1^^,^^21^ we identified most of the 13 conserved T6SS core components and two accessory proteins (TssM is absent in strain C2008000870, and TagO is lacking in ATCC 7901) in all of the sequenced strains (Figure 1A, Tables S1 and S2). Ten core and two accessory genes were typically clustered in one major T6SS operon, whereas *tssI, tagD*, and *tagH* were grouped into a separate, auxiliary gene cluster (Figure 1B). Based on these findings together, we concluded that genes encoding a Type VI secretion system are present in all known *A. aphrophilus* strains. In contrast, according to BLAST search, we found no evidence of a T6SS gene cluster present in any of the genome-sequenced A. actinomycetemcomitans strains (data not shown).

### Active expression of T6SS in *A. aphrophilus* during growth, in mono-species biofilms

**Figure 2:**
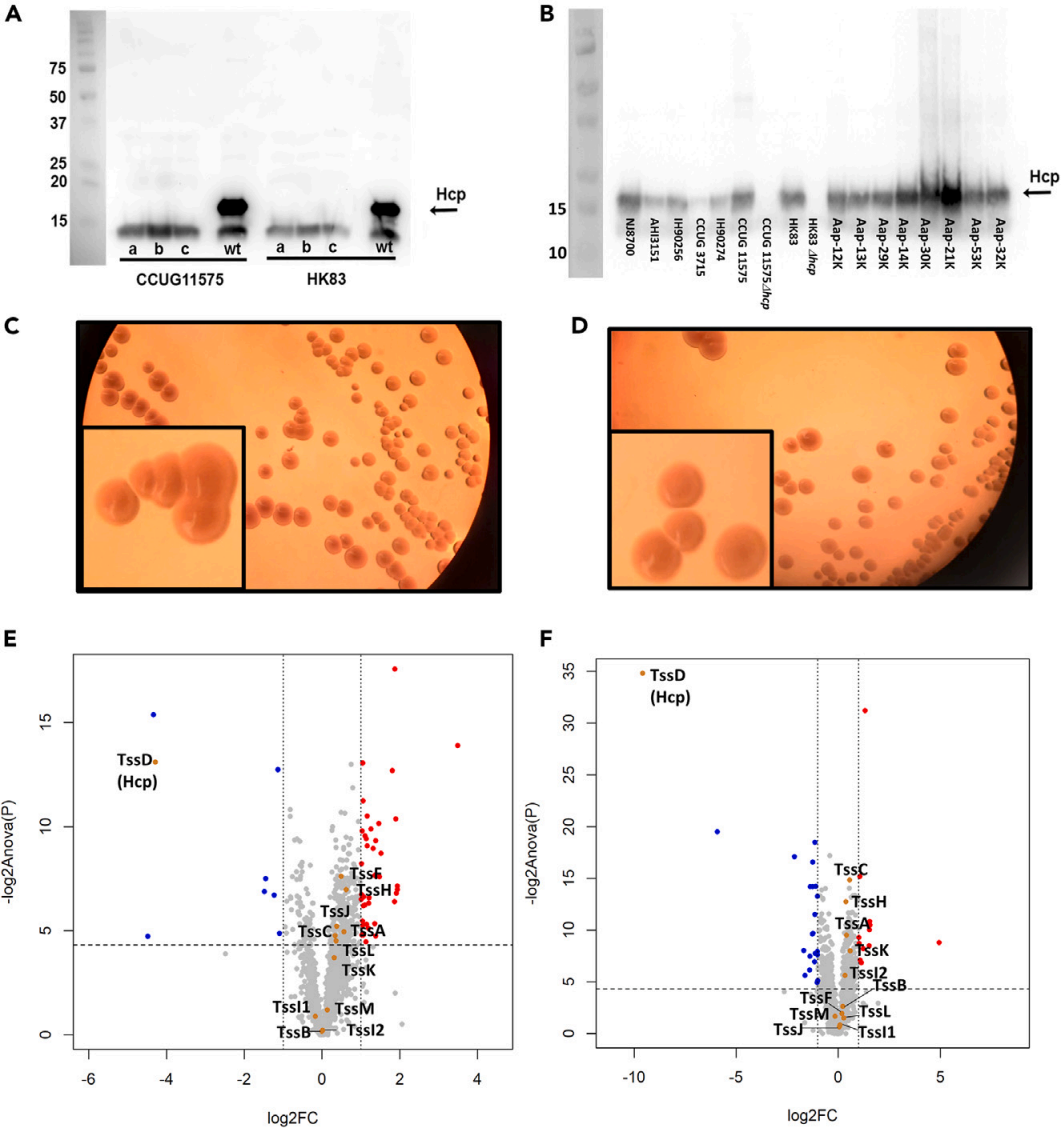
Expression of Hcp in *A. aphrophilus* strains, and regulatory trends in protein expression profiles upon gene replacement of *hcp* in mono-species biofilms (A) Western blot detection of Hcp expression in *A. aphrophilus* wildtype (wt) CCUG11575 and HK83 strains, and three independently isolated, isogenic hcpkan gene replacement mutants of each strain (a, b, and c). (B) Western blot detection of Hcp expression in *A. aphrophilus* strains. Similar colony morphology appearance of *A. aphrophilus* strain HK83. (C) and its *hcp* mutant derivative, HK83 *Δhcp*. (D) Cultivated on blood agar. The scale bar lengths correspond to 0.5 cm in all figures. (E) The Log_2_ fold change (FC) of label-free quantified full protein profiles in HK83 Δhcp compared with HK83 (*n* = 6 each). Upregulated [log2 (FC) ≥ 1, *p* ≤ 0.05] and downregulated [log2 (FC) ≤ −1, *p* ≤ 0.05] proteins were plotted in red and blue, respectively, while unregulated proteins were plotted in gray. T6SS core proteins were indicated in yellow. Vertical dashed lines represented |log2FC| = 1, and the horizontal dashed line represented *p* = 0.05. (F) The Log_2_FC of label-free quantified full protein profiles in CCUG 11575 *hcp* compared with CCUG 11575 (*n* = 6 each). Two TssI proteins (A0A3M6NIH8_AGGAP and A0A3M6PR51_AGGAP) recorded in Uniprot have been identified by us and are here indicated as Tssl1 and Tssl2.

To initially assess the functional expression of Type VI secretion in *A. aphrophilus* strains, western blot was used to monitor the production of the “tube” core protein, hemolysin co-regulated protein (Hcp; TssD), a hallmark of T6SS activity,^22^ in mono-species biofilms. Mutant derivatives with an allelic replacement of *hcp* were generated in strains HK83 and CCUG 11575, as negative controls. This revealed the expression of Hcp in all wild-type (*n* = 15), but not in the *hcp* mutant (*n* = 2) strains (Figures 2A and 2B). This is consistent with presence of active T6SS secretion during bacterial growth. As an additional negative control, also *A. actinomycetemcomitans* strain D7SS was assessed, revealing no Hcp-specific reactive band in western blot (data not shown). Moreover, inactivation of the *hcp* gene had no apparent effect on bacterial growth as evidenced via colony morphology on blood agar (Figures 2C and 2D).

### Detection of T6SS core proteins and identification of a *hcp*-affected proteins in *A. aphrophilus*, in mono-species biofilms

To assess the expression of additional T6SS core proteins in *A. aphrophilus* and their potential regulation upon *hcp* inactivation, we analyzed proteome profiles of HK83 and CCUG 11575, and their respective *hcp* mutant derivatives, respectively, cultivated in mono-species biofilms (*n* = 6, each). We detected 1517 proteins with a protein false discovery rate of 0.73% (Table S3). When applying a 2-fold change (FC) and a significance cutoff of *p* < 0.05 on protein abundance, only 3% of the proteins were significantly regulated in the *A. aphrophilus Δhcp* strains, relative to their respective parental strains (39 up-regulated ↑, and 8 down-regulated ↓ in HK83 biofilms (Figure 2E), whereas 12↑ and 23↓ in CCUG 11575 biofilms (Figure 2F). Gene ontology (GO) analysis of the T6SS-regulated revealed specific gathered functions (i.e., maximally two proteins share the same biological process) (Tables S4 and S5).

In addition to Hcp, we also detected ten additional of the 12 T6SS core proteins, including both contractile sheath proteins (TssB and TssC), the sheath recycling ATPase, TssH, the “spike protein”, TssI, three baseplate proteins (TssA, TssK, and TssF), and all three membrane complex proteins (TssJ, TssL, and TssM) (indicated as brown dots in Figures 2E and 2F)(Table S3). Of note, the expressions of other T6SS core proteins did not exhibit significant differences between the wild types and their respective *hcp* mutations (Figures 2E and 2F). In addition to our *in-silico* analysis, this finding further confirms the presence of T6SS-core proteins in *A. aphrophilus* and suggests that they are affected by the deletion of *hcp*, at least in monoculture conditions.

### *A. aphrophilus* exhibits a T6SS-dependent anti-bacterial activity against A. actinomycetemcomitans *in vitro*

**Figure 3:**
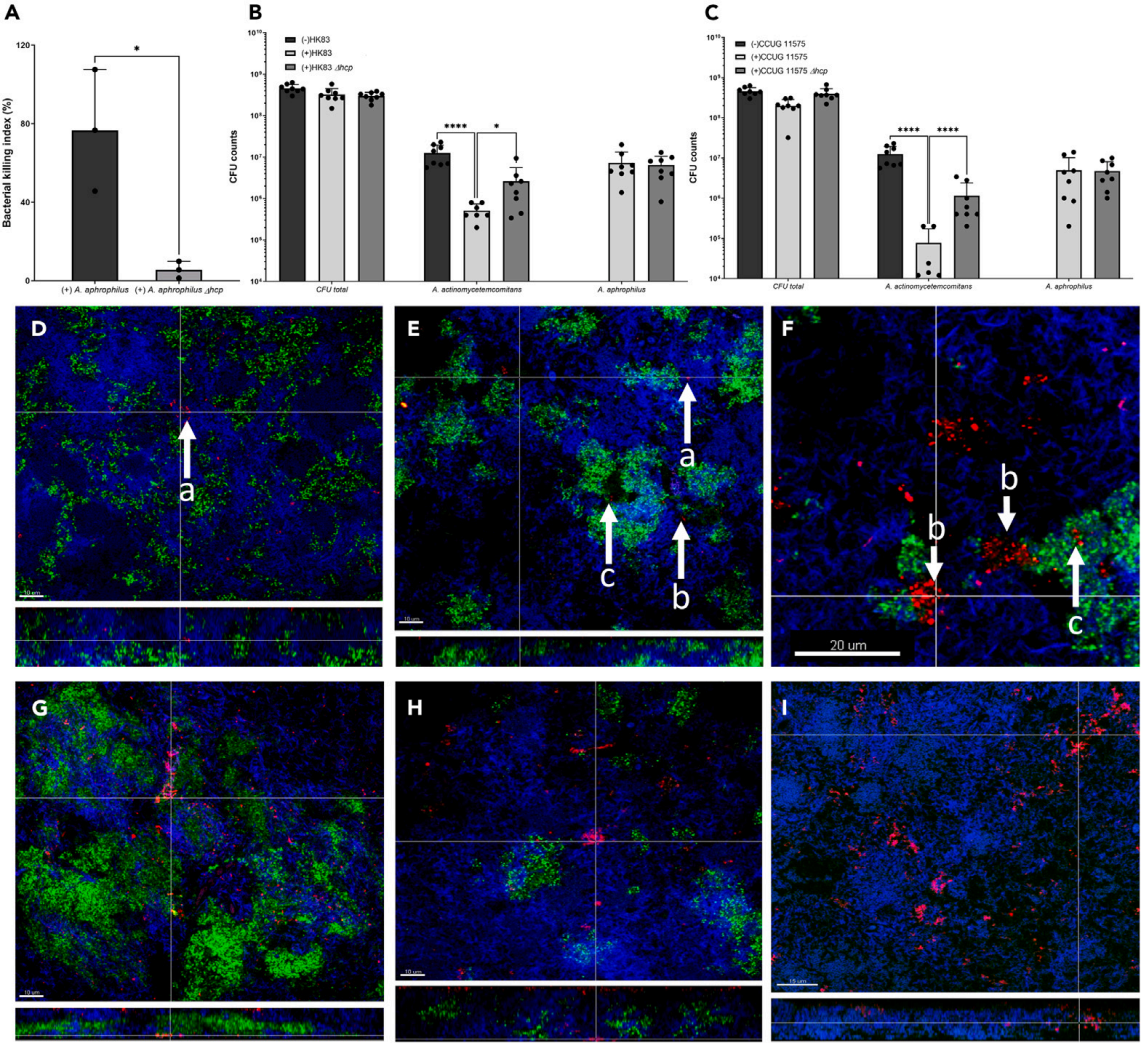
T6SS-dependent anti-bacterial activity of *A. aphrophilus* against *A. actinomycetemcomitans* The bacterial killing index (based on ratio of A. aphrophilus CFU numbers in co-culture divided by those when in monoculture) in an *A. aphrophilus* and *A. actinomycetemcomitans* co-culture environment on agar using either *A. aphrophilus* HK83 or HK83 *Δhcp*, and *A. actinomycetemcomitans* strain D7SS as prey (A). The relative cell abundances of *A. aphrophilus* and *A. actinomycetemcomitans* strain JP2, respectively, in the multi-species biofilms containing either *A. aphrophilus* strain HK83 or HK83 *Δhcp* (B), or CCUG11575 or CCUG11575 *Δhcp* (C). Quantification was performed using colony forming unit (CFU) counting from 8 biological replicates (ANOVA test: ∗*p* < 0.05). Localization of cells of *A. actinomycetemcomitans* strain JP2 (in red) and *A. aphrophilus* strains (in green) was achieved using Cyanine 3 (Cy3)-labelled *A. actinomycetemcomitans* 16S rRNA oligonucleotide probe Act639 and FAM-labeled A. aphrophilus 16S rRNA oligonucleotide probe Aaph639, respectively. This staining was performed within the multi-species biofilms: HK83 (D), CCUG11575 (E), CCUG11575 (F), HK83 *Δhcp* (G), and CCUG 11575 *Δhcp* (H). Panel (I) represents a multi-species biofilm that did not include *A. aphrophilus*. The letters on the arrows in (D) represent: a) Microcolonies of *A. actinomycetemcomitans* JP2 growing when *A. aphrophilus* was absent; b) Microcolonies of *A. actinomycetemcomitans* JP2 encounter with macrocolonies of *A. aphrophilus* in the vicinity; c) Single cells or small aggregates of *A. actinomycetemcomitans* JP2 can be identified within the biomass, embedded among microcolonies of *A. aphrophilus*. Panels D, E, G, H and I show a representative area of one disc, respectively. Panel (F) represents a magnified screenshot from an *A. aphrophilus* strain CCUG 11575-inclusive multi-species biofilm. This zoomed-in view aims to provide a closer depiction of the physical interaction between *A. aphrophilus* and *A. actinomycetemcomitans*. The scale bars for panels D, E, G, H, and I were 10, 10, 20, 10, 10, and 15 μm, respectively. Bacterial cells of additional species (blue) were counterstained with YoPro-1 iodide and Sytox Green, and their CFU data are listed in Table S6.

To test the importance of the T6SS in contact-dependent killing of competitor species, bacterial competition assays with *A. aphrophilus* strain HK83 or its hcp mutant were conducted on agar, with A. actinomycetemcomitans strain D7SS as prey. When quantifying the reduction of A. actinomycetemcomitans cell numbers using the bacterial killing index (based on ratio of *A. aphrophilus* CFU numbers in co-culture divided by those when in monoculture), a significant decrease (*p* = 0.017) was observed when co-incubated with *A. aphrophilus* HK83, in comparison to using HK83 Δhcp as a donor (Figure 3A). This reduction was substantial, with a mean ± SD of 76.6% ± 25.2% for the former and 5.6% ± 3.5% for the latter. Hence, we concluded that *A. aphrophilus* HK83 could indeed kill *A. actinomycetemcomitans* cells *in vitro*, whereas this property was lost in the *hcp* mutant, confirming the role of the T6SS in this anti-bacterial activity.

### *A. aphrophilus* specifically kills *A. actinomycetemcomitans* in a T6SS-dependent manner in oral multi-species biofilms *in vitro*

As genetically similar, these bacterial species may compete within the same oral niche. To investigate this, we utilized *in vitro* multi-species biofilm models to mimic the natural habitat for assessing the killing activity of *A. aphrophilus* against *A. actinomycetemcomitans*. This multispecies biofilm also included six additional species, representing both early and late stages of oral biofilm development. These species are *Actinomyces oris*, *Candida albicans*, *Fusobacterium nucleatum* subsp. *nucleatum*, *Streptococcus oralis*, *Streptococcus mutans*, and *Veillonella dispar*. Both *A. aphrophilus* HK83 (7.33E0^6^ ± 5.95E0^6^) and HK83 *Δhcp* (2.64E^6^ ± 2.76E^6^) survived in high numbers within the multi-species biofilms (Figure 3B and Table S6). However, when *A. aphrophilus* HK83 was present, there were significant reductions (*p* < 0.05) in the abundance of the *A. actinomycetemcomitans* strain (5.14E^5^ ± 2.10E^5^) compared to the biofilm without *A. aphrophilus* (Figure 3B) (1.26E^7^± 6.86E^6^) or one containing HK83 *Δhcp* (2.64E^6^ ± 2.76E^6^). Interestingly, HK83 *Δhcp*, which lacks Hcp expression, exhibited reduced killing of the A. actinomycetemcomitans prey strain (*p* < 0.05), with the recovered *A. actinomycetemcomitans* numbers not significantly difference to those in the biofilm without *A. aphrophilus*. Notably, neither HK83 nor HK83 *Δhcp* had a significant impact on the growth of the other species within the biofilm. The only exceptional species was *V. dispar*, which levels were reduced by 6.51 times, as compared with 24.4 times for *A. actinomycetemcomitans* in the HK83 *Δhcp* biofilm compared with the control biofilm (Table S6). Similar patterns of T6SS-dependent fitness reduction of *A. actinomycetemcomitans* were observed within CCUG 11575-containing biofilms (Figure 3C and Table S6).

Using confocal laser scanning microscopy (CLSM) in combination with *in situ* hybridization (FISH)-stained biofilms, we determined the spatial localization of selected species within the biofilms (Figures 3D–3H). In biofilms containing wildtype *A. aphrophilus*, *A. actinomycetemcomitans* was found in low abundance and primarily detected as dispersed microcolonies when *A. aphrophilus* was absent from their vicinity (Figures 3D and 3E). In contrast, densely aggregated colonies of *A. aphrophilus* were abundantly distributed throughout the entire biofilm. In addition, we observed microcolonies of A. actinomycetemcomitans embedded within a matrix, primarily located at the forefront of *A. aphrophilus* accumulations, which indicates the potential presence of contact-dependent stress (Figure 3E). Moreover, when trapped within larger *A. aphrophilus* communities, A. actinomycetemcomitans mainly formed single or small aggregates (Figure 3F), suggesting a contact-specific elimination mechanism employed by *A. aphrophilus* against A. actinomycetemcomitans within the biofilm. However, in the presence of Δhcp *A. aphrophilus* strains (Figures 3G and 3H), or in biofilms without *A. aphrophilus* (Figure 3I), *A. actinomycetemcomitans* numbers were significantly higher and the biofilm structure appeared more tightly packed, resulting in a spatially more uniform distribution of A. actinomycetemcomitans.

### T6SS-dependent metaproteome dynamics within the multi-species oral biofilms

We next assessed potential T6SS-dependent metaproteome dynamics in the co-habiting species within the multi-species biofilms containing *A. aphrophilus,* with or without Hcp expression (*n* = 4 for each group, biological replicates), to gain a concept of the potential interplay between bacteria in the oral ecosystem using our multi-species biofilm model, which is designed to replicate the composition and structures of natural dental biofilms.^19^^,^^23^^,^^24^

**Figure 4:**
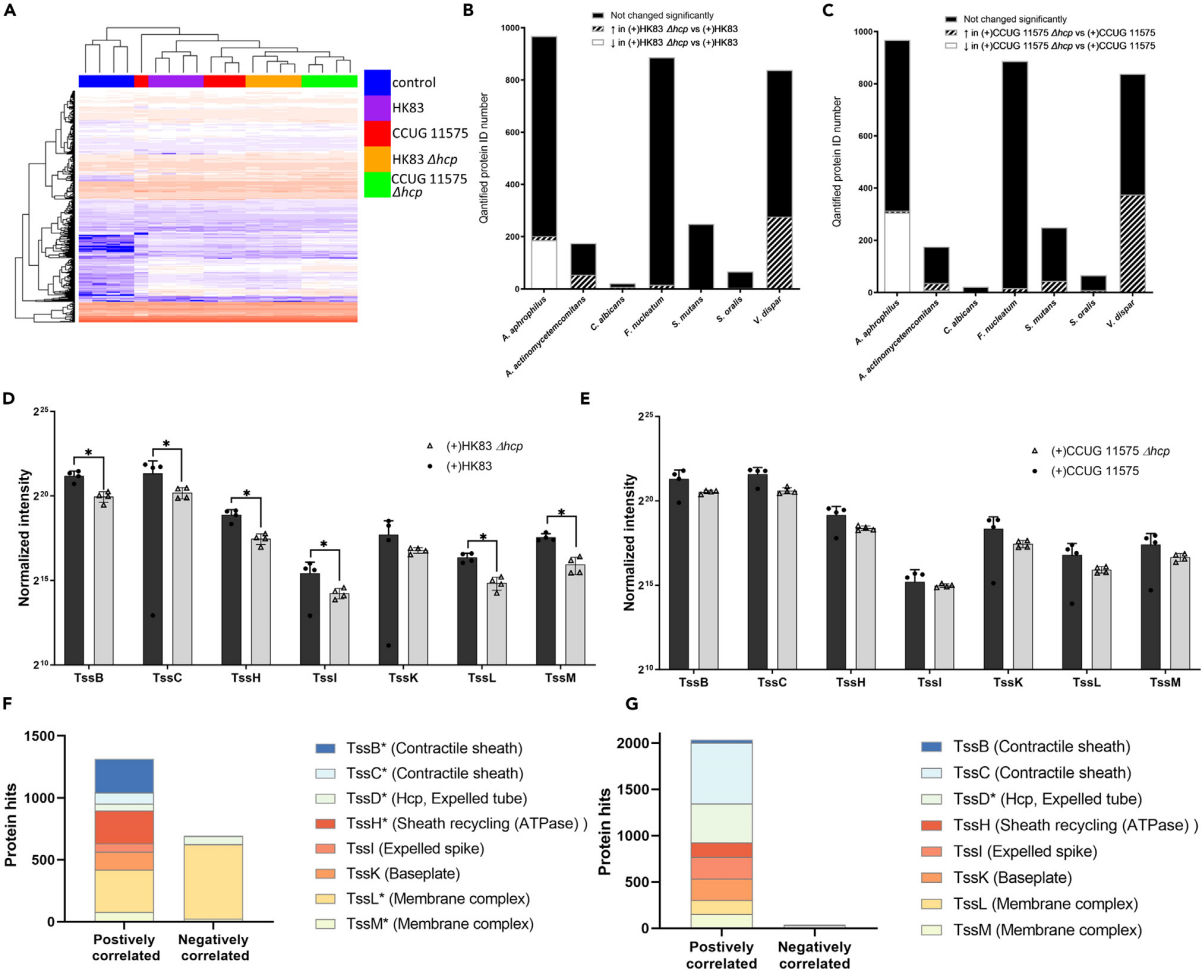
Metaproteomic shifts in multi-species biofilms (A) Protein profiles from different biofilms were compared using heatmaps based on unsupervised clustering of label-free quantitation data. The multi-species biofilms included a non-*A. aphrophilus* control (blue), or an *A. aphrophilus* strain as follows: HK83 (purple), CCUG 11575 (red), and the *hcp* mutant strains HK83 *Δhcp* (yellow), and CCUG 11575 *Δhcp* (green) as indicated. (B) The number of regulated proteins in HK83 *Δhcp* compared with HK83–included multi-species biofilms. (C) Numbers of regulated proteins in CCUG 11575 *Δhcp* compare with CCUG 11575 multi-species biofilms. *Actinomyces oris* proteins were excluded from panels B and C due to the low number of proteins identified from this species (*n* = 3). In the HK83–containing biofilms, one protein was regulated, i.e., upregulated in HK83 *Δhcp* biofilms. Conversely, no *A. oris* protein was regulated in the CCUG 11575-containing biofilms. The normalized abundance of identified T6SS core proteins (excluding Hcp) in multi-species biofilms containing HK83 *Δhcp* or HK83 (D), and CCUG 11575 *Δhcp* or CCUG (E), respectively. The number of proteins correlated to T6SS core proteins in HK83 *Δhcp* or HK83*-* included in multi-species biofilms (F). (G) CCUG 11575 *Δhcp* or CCUG 11575 included in multi-species biofilms. The results are expressed as means ± standard deviations. The asterisk (∗) denotes the core proteins that have significant differences (*p*-value<0.05 and abs(log2FC)≥2) between the *Δhcp* mutated and wild-type-included multi-species biofilms.

Label-free quantitative proteomics identified and quantified 3286 proteins (pro-FDR: 0.091%, Table S7). Unsupervised clustering analysis based on quantified protein abundance revealed that biofilms containing an *A. aphrophilus Δhcp* strain clustered together, irrespective of strain derivative used (Figure 4A), indicating a similar influence on the overall proteome composition of the biofilm. Furthermore, comparing protein abundances in biofilms containing a *Δhcp* strain instead of the isogenic wildtype, revealed a significant regulation of 564 proteins (abs(FC) > 2, *p* < 0.05) in HK83 biofilms, predominantly from *A. aphrophilus* (202) and *V. dispar* (277). In CCUG11575 biofilms, 795 proteins were significantly regulated, with 313 from *A. aphrophilus* and 375 from *V. dispar* (Figures 4B and 4C, Table S8). Unlike in mono-culture biofilm, *A. aphrophilus* proteins represented 21% of the regulated proteins in the HK83 *Δhcp* vs. the HK83 biofilms, and 32.2% in the CCUG11575 *Δhcp* vs. the CCUG 11575 biofilms (Figures 4B and 4C). This regulation in proteome composition seemed to also extend beyond known T6SS-related proteins, impacting cellular signaling such as carbohydrate processing, protein folding, and translation (Tables S8 and S9). Taken together, absence of Hcp expression in both *A. aphrophilus* strains not only altered the overall proteome composition of the entire biofilm, particularly impacting *V. dispar* but also changed various biological processes beyond the T6SS within *A. aphrophilus* itself.

### Identification of T6SS core proteins within the multi-species biofilms, and their associated proteins

In addition to TssD (Hcp), seven out of 12 other core T6SS proteins (TssB, TssC, TssH, TssI, TssK, TssL, and TssM) were identified as part of the biofilm metaproteome. When HK83 was used, a significant upregulation of five T6SS-associated proteinswas observed. In contrast, although similar regulatory patterns were observed when strain CCUG 11575 *Δhcp* was used in the biofilm compared to when the wild-type CCUG 11575, the differences were not reached definition of significant (Figures 4D and 4E).

We aimed to investigate the regulatory impact of T6SS on the entire biofilm. Since there was no inter-species functional analysis tool available, our approach relied on establishing correlations between eight core proteins with other proteins (*n* = 3201) based on their abundance changes among individual samples, without necessarily inferring a cause-and-effect relationship. We found significantly positive correlations (r > 0.7, *p*-value<0.05) of the T6SS core proteins with 582 proteins, primarily from *A. aphrophilus*, and strong negative correlations (r < −0.7, *p*-value<0.05) with 643 proteins, mainly from *V. dispar* (Table S10). All eight core proteins demonstrated robust correlations with multiple proteins, with TssB, TssH, and TssL sharing more than half of the positive correlations. TssL was particularly associated with the majority of negative correlations (Figure 4F), mainly those originating from *V. dispar*. Similar patterns were observed in the biofilms containing *A. aphrophilus* CCUG 11575 biofilms (Figure 4G).

Besides core proteins, other factors may influence T6SS expression. For instance, lipopolysaccharide (LPS) can alter the bacterial surface composition, leading to a substantial regulation of surface proteins including T6SS core proteins. Therefore, we conducted a search for all proteins identified throughout the biofilm with “lipoprotein localization to outer membrane” GO terms. Notably, we found only three proteins meeting these criteria, all exclusively *A. aphrophilus* proteins (Tables S7, S8, and S9). These proteins demonstrated a significantly decreased in HK83 *Δhcp* biofilms and display strongly positive correlations with numerous T6SS core proteins (Table S10). These proteins are outer-membrane lipoprotein LolB (A0A3M6P2B3_AGGAP), outer-membrane lipoprotein carrier protein (A0A3M6P269_AGGAP, coded by lolA), and the lipoprotein-releasing system transmembrane subunit LolE (A0A3M6PJD7_AGGAP). Similarly, two non-T6SS bacterial secretion system-related proteins, TatA (A0A0K1N244_AGGAP) and VWA domain-containing protein (A0A3M6NR98_AGGAP), were also down-regulated and highly positively correlated with different T6SS core proteins. According to the computational results from the KEGG database), A0A0K1N244_AGGAP is involved in the bacterial secretion system as an inner membrane protein for twin arginine targeting, while A0A3M6NR98_AGGAP is one of the regulatory proteins for the T6SS system (Tables S7 and S10). In CCUG 11575-containing biofilms, we also observed similar patterns in non-T6SS core secretion proteins (Table S8). This suggests that Hcp not only significantly altered abundances of T6SS-related proteins but also exerted a regulatory effect on other secretion-related and lipoprotein-localized proteins in microbial communities.

### The T6SS-dependent anti-bacterial activity of *A. aphrophilus* against *A. actinomycetemcomitans* in the *Drosophila melanogaster* infection model

**Figure 5:**
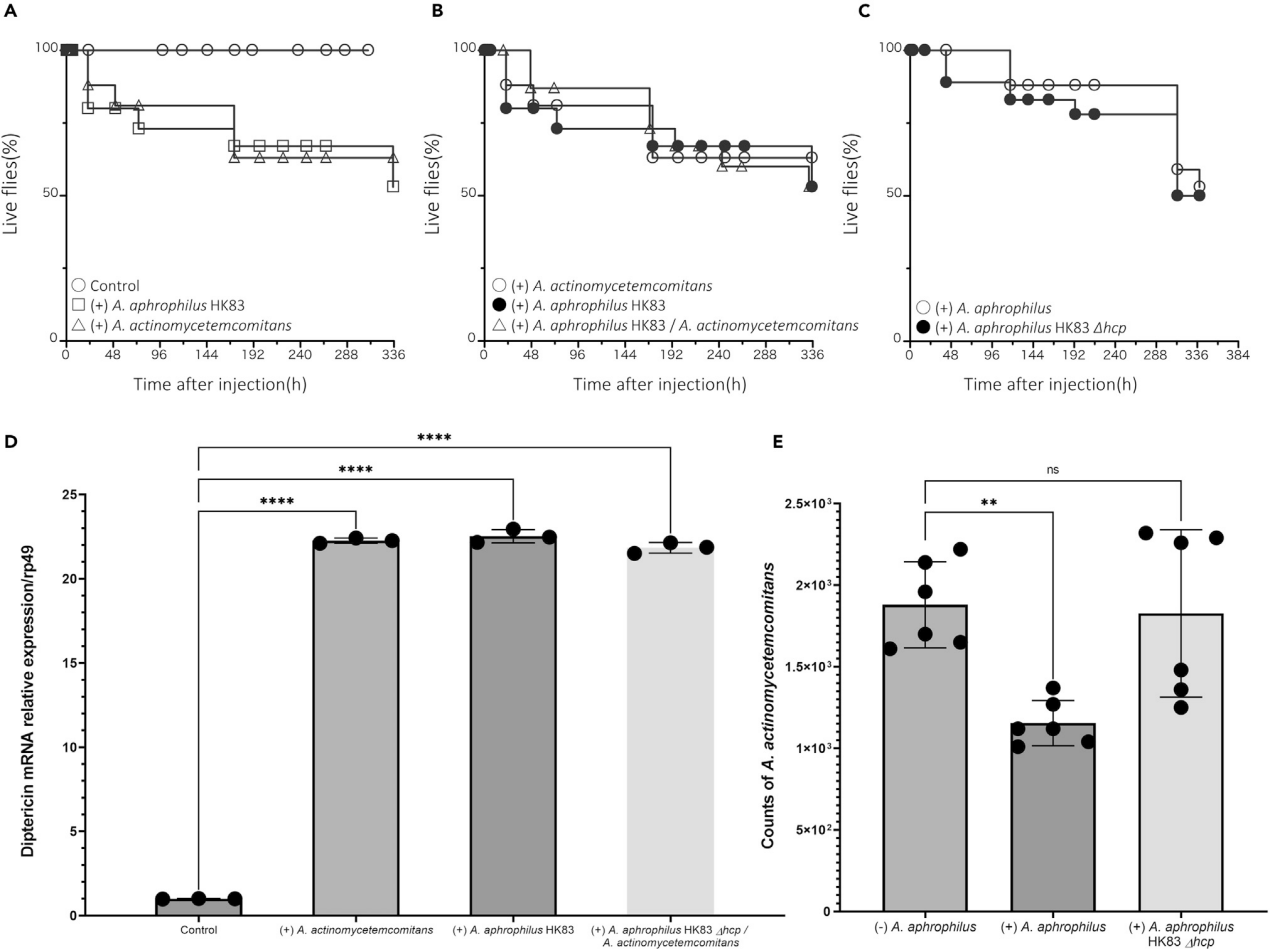
The T6SS-dependent anti-bacterial activity of *A. aphrophilus* against *A. actinomycetemcomitans* in the *D. melanogaster* model The survival (%) of flies was assessed as displayed in panels A-C as follows. Controls represent inoculation with PBS. *D. melanogaster* inoculated with the wild-type *A. aphrophilus* strain HK83, or with *A. actinomycetemcomitans* strain JP2 in monoinfection (A). Flies inoculated with *A. actinomycetemcomitans* JP2, and *A. aphrophilus* HK83 in monoinfection, and in co-infection with both strains (B). Flies inoculated with *A. aphrophilus* HK83 or with HK83 *Δhcp* in monoinfection (C). Panel D displays the abundances of *A. actinomycetemcomitans* in *D. melanogaster* 5 min after being inoculated, either with *A. actinomycetemcomitans* alone or in combination with *A. aphrophilus* HK83 or HK83 *Δhcp*. Panel E illustrates diptericin mRNA expressions in *D. melanogaster* across different groups: the none-inoculation group (control), *A. actinomycetemcomitans* alone, *A. aphrophilus* HK83 alone, or both HK83 *Δhcp* and *A. actinomycetemcomitans*. The mRNA expression levels were calculated using 2–ΔΔCt compared to the ribosomal protein rp49. The results are expressed as means ± standard deviations. Dunn’s multiple comparison test was performed to identify differences between individual time points (∗*p* < 0.001, ∗∗∗*p* < 0.00001).

*D. melanogaster* relies on humoral and cell-mediated innate rather than adaptive immunity to defend against pathogens,^25^ making it a good model to mimic the cohabiting properties of the oral environment, where innate immunity plays a unique role by triggering a crucial systemic response to protect the host and maintain homeostasis.^26^ The T6SS-dependent antagonism of *A. aphrophilus* toward *A. actinomycetemcomitans* was next assessed in a *D. melanogaster* bacterial infection model. Under our experimental conditions, the *A. aphrophilus* and *A. actinomycetemcomitans* strains tested killed approximately 20% of the flies within two days, whereas around 50% remained alive even after 14 days (Figure 5A). Notably, the survival rate of *D. melanogaster* was similarly reduced regardless both bacterial species were co-injected, or upon mono-infection (Figure 5B). Neither did lack of Hcp expression in *HK83 Δ*hcp affect the fly viability (Figure 5C). As humoral immune responses in *Drosophila* against Gram-negative bacteria are often induced through activation of the Imd pathway, inducing production of antimicrobial peptides, including diptericin, this was monitored. We observed a significant elevation of mRNA levels of diptericin in flies infected with the *A. aphrophilus* strains tested, but no significant difference between flies infected with either strain alone or with *A. aphrophilus* HK83 and *A. actinomycetemcomitans* JP2 combined (Figure 5D).

To determine the infection level in flies with *A. aphrophilus* HK83 and *A. actinomycetemcomitan*s JP2 alone or combined, we randomly selected viable flies and quantified the abundance of *A. actinomycetemcomitans* JP2 within the flies by qPCR. Lysates from uninfected flies did not yield detectable signals, suggesting endogenous bacteria did not affect the detection of these two bacteria. While levels of *A. actinomycetemcomitans* JP2 (mean ± SD 1155 ± 127) were significantly declined (*p* < 0.05) from flies once *A. aphrophilus* HK83 was co-injected, as compared with *A. actinomycetemcomitans* JP2 mono-inject group (1880 ± 241). Additionally, the *Δhcp* strains did not effectively eliminate *A. actinomycetemcomitans* JP2 (1827 ± 469) from flies, unlike the parental strain (Figure 5E). This establishes that *A. aphrophilus* effectively utilizes the T6SS to outcompete *A. actinomycetemcomitans* within this *in vivo D. melanogaster* model.

## Discussion

Here we discovered and assessed the expression of a functional T6SS in *A. aphrophilus*, an oral commensal bacterium. According to the present results, *A. aphrophilus*, as a symbiont, uses this system to specifically target a pathobiont, i.e., its close relative *A. actinomycetemcomitans*, a species implicated in infective endocarditis and periodontitis among young individuals.^13^ To the best of our knowledge, this is the first commonly carried oral bacterium found to possess the T6SS secretion system. The comprehensive investigation of symbiont-pathobiont antagonism suggest that the *A. aphrophilus* T6SS serves a crucial role in the competitive activity of this species against *A. actinomycetemcomitans* in niches where the two related, yet strongly antagonistic, species may coexist.

Although there are free-living bacteria with the T6SS, T6SS is more common among complex microbial communities.^27^ Oral biofilms are highly diverse multi-species microbial communities encompassing more than 700 different bacterial species.^28^ *A. aphrophilus* and *A. actinomycetemcomitans*, show high genetic similarity (approximately 80% in gene content),^9^ suggesting their evolution from a common ancestor to establish a competitive relationship within the oral ecological habitat. Supporting this speculation, our recent assessment of the Maasai population supports this notion as, bacterial loads of *A. actinomycetemcomitans* and *A. aphrophilus* were inversely related in dental biofilms.^29^ Multi-species biofilm model was designed to replicate the composition and structures of natural dental biofilms,^19^^,^^23^^,^^24^ and demonstrated the proximity of these two species within the microbial community. Both *Aggregatibacter* species possess strong aggregative properties,^30^^,^^31^ which may facilitate the activity of a contact-dependent T6SS.^32^

The T6SS nanomachine delivers effector proteins targeting neighboring “prey” bacterial cells.^33^ The T6SS and its components were first named in *Vibrio cholerae*,^22^ however, T6SS-encoding genes were discovered earlier^34^ and believed to be widely distributed in nearly 25% of Gram-negative bacteria.^33^^,^^35^ A T6SS unit typically consists of 13 core protein components and a few accessory proteins, named using a unifying “Tss” and “Tag” nomenclature.^1^^,^^21^ Our *in silico* searches revealed genes encoding all 13 core components of the T6SS, along with three major accessory proteins, in most *A. aphrophilus* genomes available in the NCBI database. Evidently, expressing a T6SS is energetically expensive and tightly regulated in bacteria.^36^ In *V. cholerae*, the expression of TssD was halted at stationary phase^37^ as Hcp accumulation inhibits its own synthesis, and that of other T6SS components.^38^ However, except Hcp, we did not observe a complete or significant reduction in the expression of any of the other T6SS core components during mono-culture conditions. This discrepancy may be attributed to the apparent absence of the bacterial enhancer binding protein, VasH in *A. aphrophilus* genomes. In *V. cholerae*, VasH is encoded within the large virulence-associated secretion (VAS) cluster operon, which also encompasses several T6SS core components. Some of these core proteins, particularly TssM (VasK), have been shown to inhibit T6SS activity and the eradication of certain bacterial competitors, as well as host cells, in *Drosophila* models.^39^ Therefore, VasH is crucial for the Hcp-dependent regulation of T6SS component expression.^38^ VasH is a σ^54^-dependent-transcriptional activator in *V. cholerae*,^40^ suggesting the possibility that the T6SS of *A. aphrophilus* might be controlled within such a regulon as well. This remains to be experimentally assessed. Interestingly, in contrast to the mono-culture biofilms, we observed elevated levels of six out of eight T6SS core proteins in multi-species biofilms containing a wild-type *A. aphrophilus* strain compared to those with *hcp* mutant derivatives. Moreover, many non-T6SS related proteins were also associated with T6SS core proteins in multi-species biofilms, including A0A3M6NR98_AGGAP and all three lipoproteins A0A3M6P269_AGGAP, A0A3M6P2B3_AGGAP, and A0A3M6PJD7_AGGAP. The former one is homologous to PpKA in *P. aeruginosa*, known for its crucial role in T6SS regulation.^41^ Earlier studies have demonstrated that the depletion of lipopolysaccharide can alter the composition of the bacterial surface and subsequently lead to a substantial decrease in the expression of the Hcp homolog.^42^ Importantly, our mono-species experiment showed no regulation of these proteins, in contrast to the results observed in the multi-species model where regulation and association were evident. This suggests that the presence of other species in the multi-species environment can crucial for triggering Hcp-dependent regulation in T6SS core components and other proteins, potentially through quorum sensing mechanisms. In *V. cholerae*, quorum-sensing factor regulators such as LuxO,^43^ HapR,^37^ and quorum-regulatory small RNAs^44^ exhibited an apparent regulation on the T6SS expression. Although *A. aphrophilus* lacks the LuxO-HapR cascade-related quorum sensing regulation found in *V, cholera*, our studies discovered the LuxS production and other transport system ATP-binding proteins related to LuxS/AI-2 quorum sensing. Overall, the regulation of T6SS core proteins emphasizes the significance of T6SS regulation in a multi-species environment, likely mediated by quorum sensing mechanisms.

Furthermore, we used the *D. melanogaster* infection model to test whether T6SS-mediated pathobiont-symbiont interactions take place *in vivo.*^26^^,^^39^^,^^45^ It is a well-equipped model to study many aspects of host-bacterial interactions. Interestingly, we found that inoculated flies with all tested species, either alone or in combination with *A. aphrophilus*, was sufficient to kill the host independent of T6SS. This may imply that both species may use additional virulence factors to kill the host in a T6SS-independent manner. Finally, we noted that the antimicrobial response of *Drosophila* is nonspecific across both species, as evidenced by the absence of differential expression in diptericin. This finding aligns with previous studies by where non-specific Gram-negative bacteria-induced diptericin upregulation was documented. Despite, potent antimicrobial response, the elimination of *A. actinomycetemcomitans* by *A. aphrophilus* in *Drosophila* strongly support a role of *A. aphrophilus* T6SS as a highly functional molecular tool that enhances the fitness and competitive advantage of this species within their co-existing niches.

In summary, we demonstrate first time that oral symbiont *Aggregatibacter aphrophilus* possesses a T6SS and can eliminate its close relative oral pathobiont *Aggregatibacter actinomycetemcomitans* using its T6SS. The potential effect this system may have on *V. dispar* will be investigated in further studies. These findings bring newer the anti-bacterial prospects of symbionts against cohabiting pathobionts while introducing presence of an active T6SS in the oral cavity. Apart from being a seminal discovery in the field of oral ecology and beyond, it may represent an important step toward a more thorough understanding of bacterial competition strategies within complex ecosystems where they are present. Future studies on potential antibacterial effectors of the T6SS of *A. aphrophilus* will be pursued, and it is plausible that exploitation of the T6SS of *A. aphrophilus* could be used in future oral microbiome therapies as well.

### Limitations of the study

A limitation of the present study is that we do not have data on the role of T6SS on the regulation of the host response, but we plan to address that in future studies.

## STAR★Methods

### Key resources table

**Table:** 

REAGENT or RESOURCE	SOURCE	IDENTIFIER
**Antibodies**		

Rabbit polyclonal antiserum specific for *V. cholerae* Hcp	Ishikawa et al.^37^	RRID: AB_2313773
Anti-rabbit horseradish peroxidase - conjugate	Jackson Immuno Research, Newmarket, UK	RRID: AB_2313773

**Bacterial and virus strains**		

*A. aphrophilus* strains HK83	Culture Collection University of Gothenburg (CCUG)	CCUG 49494
*A. aphrophilus* strains CCUG 11575	CCUG	CCUG 11575
*A. aphrophilus* strains NJ8700	CCUG	NJ8700
*A. aphrophilus* strains Aap-4K	Isolated from a patient^29^^,^^46^	Aap-4K
*A. aphrophilus* strains Aap-12K	Isolated from a patient^29^^,^^46^	Aap-12K
*A. aphrophilus* strains Aap-13K	Isolated from a patient^29^^,^^46^	Aap-13K
*A. aphrophilus* strains Aap-21K	Isolated from a patient^29^^,^^46^	Aap-21K
*A. aphrophilus* strains Aap-29K	Isolated from a patient^29^^,^^46^	Aap-29K
*A. aphrophilus* strains Aap-30K	Isolated from a patient^29^^,^^46^	Aap-30K
*A. aphrophilus* strains Aap-32K	Isolated from a patient^29^^,^^46^	Aap-32K
*A. aphrophilus* strains Aap-53K	Isolated from a patient^29^^,^^46^	Aap-53K
*A. aphrophilus* strains AHI-3151	Isolated from a patient^47^^,^^48^	AHI-3151
*A. aphrophilus* strains IH-90256	Isolated from a patient^47^^,^^48^	IH-90256
*A. aphrophilus* strains IH-90274	Isolated from a patient^47^^,^^48^	IH-90274
*A. actinomycetemcomitans* strain D7SS	Isolated from a patient^49^	D7SS
*A. actinomycetemcomitans* strain JP2	Collection from division of Clinical Oral Microbiology and Immunology, university of Zurich (OMZ)	OMZ 295
*Actinomyces oris*	OMZ	OMZ 745
*Candida albicans*	OMZ	OMZ 110
*Fusobacterium nucleatum subsp. nucleatum* KP-F2	OMZ	OMZ 598
*Streptococcus oralis* SK248	OMZ	OMZ 607
*Streptococcus mutans* UA159	OMZ	OMZ 918
*V. dispar* ATCC 17748T	OMZ	OMZ 493

**Chemicals, peptides, and recombinant proteins**		

Clarity™ Western ECL Substrate	Bio-Rad	Cat#1705062

**Deposited data**		

Proteomic raw files	ProteomeXchange	PXD042723

**Experimental models: Organisms/strains**		

*Drosophila melanogaster*	Kyorin-Fly, Kyorin University, Tokyo, Japan	Line Oregon R

**Oligonucleotides**		

The *A. actinomycetemcomitans*-specific primers 5’-GAACCTTACCTACTCTTGACATCCGAA-3(forward) and 5’-TGCAGCACCTGTCTCAAAGC-3’ (reverse)	Choi et al.^50^	N/A
Cyanine 3 -labelled *A. actinomycetemcomitans* 16S rRNA oligonucleotide probe Act639 5’-CTCCAGACCCCCAGTATG-3’	Thurnheer and Belibasakis^24^	Act639
FAM-labelled *A. aprophilus* 16S rRNA oligonucleotide 5’-CTCTAGACCCCCAGTCTG-3’	This work	Aaph639

**Software and algorithms**		

Quantable packages		https://github.com/protViz/quantable
Progenesis QI for proteomics		https://www.nonlinear.com/progenesis/qi-for-proteomics/

### Resource availability

#### Lead contact

Further information and requests for reagents may be directed to and will be fulfilled by the lead contact, Jan Oscarsson (jan.oscarsson@umu.se).

#### Materials availability

All *A. aphrophilus* strains generated in this study are available upon request.

#### Data and code availability

Data: The mass spectrometry proteomics data have been deposited to the ProteomeXchange Consortium via the PRIDE^51^ partner repository with the dataset identifier PXD042723.

### Experimental model and study participant details

*A. aphrophilus* strains were collected from Umeå University. Mutant derivatives were constructed there, and mono-species biofilm-related experiments were also performed in Umeå University. Multi-species biofilm-related experiments took place at University of Zurich, while Drosophila-related experiments were conducted at Sapporo Medical University. Protein extraction and proteomic experiments were carried out at the Functional Genomics Center Zurich. For additional details, please refer to the method details section.

### Method details

#### Ethics considerations

All procedures were conducted in accordance with the guidelines of the local ethics committee at the Medical Faculty of Umeå University, which are in compliance with the Declaration of Helsinki (64th WMA General Assembly, Fortaleza, October 2013). The *Drosophila* experiment plan was approved by the ethics review committee of Kanazawa University (#6-1790).

#### Screening for Type VI secretion systems encoded in *Aggregatibacter aphrophilus* genomes

To identify T6SS component protein sequences, BLAST searches^52^ were conducted against whole genome sequence assemblies of *A. aphrophilus* strains (n=18 in December 2022) available in the NCBI database. These previously sequenced genomes and their GenBank accession numbers are listed in Table S1. Selected additional, *A. aphrophilus* strains were assessed by PCR, using the primer pairs *hcp*_F1 (5′-CCTACACCAGCGTTTATTTC-3′) and *hcp*_R1 (5’-CTGAGGTTTACGCCAGTC-3’), amplifying an internal fragment of the *hcp* gene.

#### Bacterial strains and growth conditions

The naturally genetic competent *A. aphrophilus* strains HK83 (CCUG 49494), and CCUG 11575 were originally sampled from saliva, and brain abscesses, respectively,^53^ and thereafter transformed into a V factor-independent growth phenotype.^46^ Mutant derivatives HK83 *hcp*::*kan* [Km^r^] and CCUG 11575 *hcp*::*kan* [Km^r^] were generated in the present work. CCUG 3715 and NJ8700 are reference strains of *A. aphrophilus*.^10^^,^^53^ Strains AHI-3151, IH-90256, and IH-90274 are part of the collection of clinical isolates of *A*. *aphrophilus* in our laboratory, made by Dr. Sirkka Asikainen.^47^^,^^48^ The *A. aphrophilus* strains Aap-4K, Aap-12K, Aap-13K, Aap-21K, Aap-29K, Aap-30K, Aap-32K, and Aap-53K were collected in a field study.^29^^,^^46^ *A. actinomycetemcomitans* strain D7SS is a naturally genetic competent, smooth-colony derivative of D7S (serotype a), which was originally isolated from a patient with aggressive periodontal disease.^49^ The *A. actinomycetemcomitans* and *A. aphrophilus* strains were routinely cultivated in air supplemented with 5% CO_2_ at 37°C as previously described,^54^ on blood agar plates (5% defibrinated horse blood, 5 mg hemin/l, 10 mg Vitamin K/l, Columbia agar base). Alternatively, for transformation assays, the *A. aphrophilus* strains were grown on Trypticase soy broth (TSB) supplemented with 0.1% yeast extract, 5% heat-inactivated horse serum, and 1.5% agar (sTSB agar). *Escherichia coli* laboratory strain DH5α^55^ was used for maintenance of the plasmid pUC4K, and was cultured aerobically at 37°C in Luria-Bertani (LB) broth, or on LB broth solidified with 1.5% (w/v) agar. When needed, growth media was supplemented with 100 μg/ml (final concentration) kanamycin, rifampicin, or streptomycin.

#### Construction of *A. aphrophilus* gene replacement mutant derivatives

A PCR-based approach following standard cloning procedures^56^ was used to construct the *hcp* gene replacement mutants in naturally competent strains of *A. aphrophilus*, i.e., HK83 and CCUG 11575. Whole genome sequence data of these strains were available via GenBank (assembly accession numbers GCA_003130375.1, and GCA_003703745.1, respectively), and used as references in oligonucleotide synthesis for gene replacement. In brief, PCR fragments flanking the *hcp* gene were amplified. An upstream, 856-bp fragment, was generated using the primers *hcp*_F2 (5’-CGAGCGCAGGATTATAGCAGCT-3’) and *hcp*_R2 (5’- AAACGCTGGT**GGATCC**ATAGAATTCTC-3’), and a downstream, 1,024-bp fragment was generated using the primers *hcp*_F3 (5’-GATGACTGGC**GGATCC**CTCAGGTT-3’) and *hcp*_R3 (5’-CACCGCTTGTGTATTGGCAGTGGC -3’). The PCR primers contained a BamHI restriction site where indicated (underlined bold text), allowing ligation of the PCR fragments to flank the kanamycin resistance gene from pUC4K.^57^ Ligation products were then used to transform HK83 and CCUG 11575 on agar plates using procedures described earlier.^49^ Confirmation of allelic replacements and the orientation of the inserted resistance cassette were done by DNA sequencing and PCR. For this we used the *hcp* upstream and downstream oligonucleotide primers, respectively, in combination with a primer specific for the kanamycin resistance cassette (H7R: 5ʹ-GGACGGCGGCTTTGTTGAATAAATCG-3ʹ).

#### SDS-PAGE and western blot analysis

The SDS-Page and Western blot were used to detect expression of Hcp in the mono-species biofilm on the blood agar plates as described previously.^58^^,^^59^ For the Western blot, we used a rabbit polyclonal antiserum specific for *V. cholerae* Hcp^37^ (final dilution 1:5,000). The Hcp proteins of *V. cholerae* and *A. aphrophilus* exhibit approximately 70 % amino acid sequence identity. As a secondary antibody, anti-rabbit horseradish peroxidase (HRP)-conjugate was used (Jackson ImmunoResearch, Newmarket, UK) (1:10,000). Immunoreactive bands were visualized using Clarity™ Western ECL Substrate (Bio-Rad) and the ChemiDoc™ XRS+ System (Bio-Rad).

#### Bacterial killing assay of interbacterial virulence

Competition experiments on agar were carried out essentially as described earlier.^60^ Spontaneous rifampicin- and streptomycin-resistant derivatives of the *A. aphrophilus* and *A. actinomycetemcomitans* model strains, respectively, and *A. aprophilus hcp* mutants, were isolated for these experiments. Donor bacterial cells (OD_600_ = 1.7 in TSB) were mixed with recipient bacterial cells (OD_600_ = 1.3 in TSB) at a ratio of 3:1. Aliquots of 40 μl were spotted on blood agar plates and incubated overnight at 37°C (5% CO_2_). Cells were then harvested and competition analyzed. Colony-forming units (CFUs) of the donor and recipient were enumerated on blood agar plates supplemented with rifampin and streptomycin, respectively.

#### Multi-species biofilm formation and harvesting

A seven-species biofilm was cultivated as previously described.^61^ It contained *A. actinomycetemcomitans* strain JP2 (OMZ 295), *Actinomyces oris* (OMZ 745), *Candida albicans* (OMZ 110) *Fusobacterium nucleatum subsp. nucleatum* KP-F2 (OMZ 598), *Streptococcus oralis* SK248 (OMZ 607), *Streptococcus mutans* UA159 (OMZ 918), and *V. dispar* ATCC 17748T (OMZ 493) . Two modified biofilms, with an additional *A. aphrophilus* strain, i.e., HK83 (CCUG 49494) or HK83 Δ*hcp*, were also developed in parallel. Briefly, 200 μl of each species at densities of OD_550nm_ = 1.0 ( ± 0.05) were aliquoted on the surface of the hydroxyapatite (HA) dish and anaerobically incubated for 64 h. During the incubation, the cultivated medium was replenished at 16 h and 40 h, while the HA dishes were dip-washed in 0.9% w/v NaCl at 16 h, 20 h, 40 h, 44 h, 48 h, and 64 h. After the incubation, biofilms were collected in 0.9% w/v NaCl and processed for CFU count or incubated at -80°C for proteomic analysis. Biofilm supernatant was filtered with a 0.2 μm syringe filter (Acrodisc) before being stored at -80°C for further usage.

#### Protein extraction and clean up

Proteins from biofilm (n=4 for each) were extracted and lysed in Microcon YM-30 centrifugal filter unit (Millipore) following the protocol described previously.^20^ In brief, 20 μg of biofilm extracts were denatured with 8 M urea buffer (in 100 mM Tris/HCl buffer, pH 8.2), alkylated with 0.05 M iodoacetamide, washed by 0.5M NaCl, and digested by trypsin (Sigma-Aldrich) in an enzyme/protein ratio = 1:50 w/w overnight at room temperature. The digested solutions were then purified with StageTips (200 μL tip with a C18 disk core (Thermo Scientific)). Proteins from co-cultured samples were digested and cleaned using the in-StageTip (iST) sample preparation kit (PreOmic). Cells seeded in 24-well plates collected from co-cultured assay were washed with ice-cold phosphate-buffered saline (PBS), lysed in lysis buffer from the iST sample, and then removed by cell scraper. 50 μg of cured extracted proteins were collected and processed following the manufactoring protocol of the iST kit. Two snap-frozen flies were homogenized in 140 μL 8 M urea buffer (in 100 mM Tris/HCl buffer, pH 8.2) by a 5 mm stainless steel bead (Qiagen). The bead milling was repeated three times, each for five minutes at 30 Hz in a Tissuelyser II lysis (Qiagen). Then, 50 ug of extracted *Drosophila* proteins were digested using the iST sample preparation kit (PreOmic). The purified peptides were dried in a Speedvac (Thermo Savant SPD121P, Thermo Scientific), reconstituted in 50mM NH4FA (pH 10 with NH_4_OH) stock solution, then loaded onto StageTips to clean up the salt. The bound peptides were then eluted with 5, 10 and 30 % of acetonitrile (ACN) solutions (in 50mM NH4FA), respectively. Both purified and fractionated peptides were eventually dried in a Speedvac (Thermo Savant SPD121P, Thermo Scientific) and stored at –20°C until further usage.

#### LC-MS/MS analysis

Frozen peptides were reconstituted in 3% ACN and 0.1% formic acid. A pooled sample was created by mixing 1 μL of each sample for every experiment. All samples in the same experiment were subjected together in a random order to an Orbitrap Fusion mass spectrometer (Thermo Fisher Scientific) for proteomic analysis as described previously^62^ with the modification described below. In brief, peptides were first separated using a Thermo Scientific EASY-nLC 1200 system (Thermo Fisher Scientific) coupled to a 15 cm-long and 75 μm-diameter silica emitter as well as a ReproSil-Pur C18-AQ 120 A and 1.9 μm resin (Dr Maisch HPLC GmbH). A three-liner gradient of acetonitrile/water (containing 0.1% formic acid, at a flow rate of 300 nL/minute), first from 2% to 30% acetonitrile in 60 min, second from 30% to 97% in 10 minutes, then 97% for 10 min, was applied. The mass spectrometer was set in a data-dependent manner with an automatic switch between MS and MS/MS using the Xcalibur software package (Thermo Fisher Scientific). A mass range of 300–1500 m/z was selected for the Orbitrap analyzer.

#### Database generation

Two separate in-house databases were constructed, i.e., one for the mono-species biofilm (https://fgcz-ms.uzh.ch/FASTA/p2953_db5_d_Aaphro_20210803.fasta) and another for the multi-species biofilm (https://fgcz-ms.uzh.ch/FASTA/p2953_db5_d_Aaphro_20210803.fasta). Each database had a 260-sequences MS contaminants database (https://fgcz-proteomics.uzh.ch/fasta/fgcz_contaminants_20100901.fasta), and non-redundant databases containing all strains belonging to the target species sourced (i.e., *A. aphrophilus* for mono-species biofilms, and *A. oris*, *A. aphrophilus*, *A. actinomycetemcomitans*, *C. albicans*, *F. nucleatum*, *Homo sapiens*, *S. cerevisiae*, *S. mutans*, *S. oralis*, and *V. dispar* for mono-species biofilms.) from Uniprot. In addition, reverse sequences were included as decoys to facilitate the calculation of the false discovery rate. The Uniprot proteome identifiers (UPID) and the NCBI taxon identifiers for each database were listed in Table S11.

#### Functional analysis of proteins in biofilms

The Gene Ontology (GO) information of all identified proteins was downloaded from UniProt (accessed on September 13th, 2020, for multispecies biofilm and August 31st, 2021, for mono-species biofilm) to summarize the regulated proteins. The predicted function of *A. aphrophilus* was constructed based on the computational results from the KEGG database (accessed on December 19th, 2022).

#### Protein quantification

Progenesis QI for proteomics (version 4.1 Nonlinear Dynamics) was used for label-free quantification as described previously.^62^ In brief, all raw files from an experiment were aligned with its corresponding pooled sample as an alignment reference. All alignment results were thoroughly reviewed to verify that the alignment scores exceeded 50% before proceeding with peak picking. After peak picking, the normalization results were assessed to ensure that the difference between the largest and smallest values was within a 10-fold range. Any samples that failed to meet the expected alignment scores or normalization criteria were considered outliers and excluded from further analysis (not included in the experiment). Subsequently, peptides with charges 2+, 3+, or 4+ were selected for export as a mascot generic file (mgf). The top 5 MS/MS spectra per feature were chosen for export, and a maximum limit of 200 ions was enforced to control the fragment ion count. Deisotoping and charge deconvolution were included as essential steps in the data processing pipeline. The resulting mgf files were exported and searched using Mascot (version 2.4.1, Matrix Science) using the following parameters precursor tolerance: ± 10 ppm; fragment ion tolerance: ± 0.6 Da; Instrument type: LTQ-ORBI-Default; enzyme: trypsin; maximum missed cleavages: 2; fixed medication: Carbamidomethyl (C); variable modification: deamidated (NQ), oxidation (M) and acetyl (Protein N-term) against their corresponding databases.

The spectrum reports of the search result were generated by Scaffold v4.0 (Proteome Software) with a threshold of protFDR of 1%, minimal one peptide and pepFDR of 0.5% for biofilm and co-culture experiment, while the threshold for drosophila experiment was protFDR of 5%, minimal one peptide and pepFDR of 5%. These reports were imported in Progenesis QI for Proteomics for identifying the quantified proteins. For *Drosophila* experiments, each of the three fractions (i.e. Eluted from 5, 10 and 30 % of ACN) was first analysed separately and later recombined using the “combine analysed fractions” feather in Progenesis QI for Proteomics.

Only proteins with at least two peptides identified were considered in the study.

#### Data clustering and heat maps for regulated proteins

The R software (R: A Language and Environment for Statistical Computing, R Development Core Team) in particular the Quantable packages (https://github.com/protViz/quantable) were used to generate to heatmaps and unsupervised clustering analysis of quantified proteins.

### Quantification and statistical analysis

The protein quantification data are derived from normalized protein abundances between the given two conditions within each experiment. The significance of differences for a specific protein between strains was calculated using a two-tailed Student's t-test in Progenesis QI. Proteins exhibiting an absolute log2-fold change >1 and a Student's t-test p-value < 0.05 were considered as truly regulated. Additionally, multiple comparison (q-value) and power analysis for each protein were provided using Progenesis QI, and these results are included in the corresponding supplementary tables.

#### Image processing

Images for Fig.s were assembled using Adobe Photoshop CS6 or Microsoft PowerPoint. The correlations between T6SS core proteins and other regulated proteins were generated with the R software in particular the corrplot packages (https://cran.r-project.org/web/packages/corrplot/index.html). The significance levels of the correlations were set to p<0.05.

#### Confocal laser scanning microscopy and image analysis

Confocal laser scanning microscopy (CLSM) and image analysis were employed to evaluate the localization pattern of *A. aphrophilus* and *A. actinomycetemcomitans* within the biofilm structure. In this study, biofilm-containing discs were prepared and subjected to fluorescence *in situ* hybridization (FISH) using a Cyanine 3 (Cy3)-labelled *A. actinomycetemcomitans* 16S rRNA oligonucleotide probe Act639 (5’-CTCCAGACCCCCAGTATG-3’),^24^ and a FAM-labelled *A. aprophilus* 16S rRNA oligonucleotide probe Aaph639 (5’-CTCTAGACCCCCAGTCTG-3’) (this work). The FISH-stained discs were counterstained with YoPro-1 iodide and Sytox Green, following the previously described protocol.^63^ Visualization of the stained samples was performed using a Leica SP-5 microscope equipped with a resonant scanner system (8000 Hz), an argon laser (excitation wavelengths: 458 nm, 476 nm, 488 nm, 496 nm, and 514 nm), and a helium-neon laser (excitation wavelengths: 561 nm, 594 nm, and 633 nm). Filters were set to detect green fluorescence from the YoPro-1 iodide and Sytox Green mixture (500-540 nm) and Cy3 (570-630 nm). A glycerol immersion objective with a numerical aperture (NA) of 1.3 and 63x magnification was used for image acquisition. The acquired images were further processed using Imaris 7.4.0 software (Bitplane) to reconstruct the biofilm structure virtually. This processing allowed for a comprehensive analysis of the localization pattern of *A. actinomycetemcomitans* within the biofilm.

#### *D. melanogaster* stocks and bacterial infection assays

The *Drosophila* lines *Oregon R* (Kyorin-Fly, Kyorin University, Tokyo, Japan) was used in this study and microinjection of the bacterial strains into a hemocoel was carried out as we reported previously.^64^ Briefly, male flies, 3∼7 days after eclosion (15 to 20 flies per vial, 1 to 3 vials in each experiment), were anaesthetized with CO_2_ and injected with 100 nL of PBS with or without (as control) 1/500 loop of bacteria using a nitrogen gas-operated microinjector (Narishige, Tokyo, Japan). Flies that had received infections were maintained at 29°C with the usual food until they were subjected to the analysis. The number of dead flies was counted to evaluate the virulence of the bacteria.

#### Real-time quantitative PCR (qPCR)

The abundance of *A. actinomycetemcomitans* within the flies was determined by qPCR as described previously.^65^ In brief, bacteria-injected flies were first homogenized with micromixer pestles and their genomic DNA was extracted using the GenElute bacterial genomic DNA kit (Sigma-Aldrich). The extracted DNA was subjected to quantitative PCR using THUNDERBIRD SYBR qPCR Mix (Toyobo, Osaka, Japan) and Mx5005p (Agilent, CA). The *A. actinomycetemcomitans*-specific primers 5’-GAACCTTACCTACTCTTGACATCCGAA-3’ (forward) and 5’-TGCAGCACCTGTCTCAAAGC-3’ (reverse)^50^ targeting the 16S ribosomal RNA were used. The bacterial numbers per fly were calculated using standard curves generated with *A. actinomycetemcomitans* DNA extracted from known cell numbers as described previously.^62^

## Supplemental information

Table S1. The whole genome sequences of A. aphrophilus (n = 25) available in the National Center for Biotechnology (NCBI) database, related to Figure 1A

Table S2. The list of A. aphrophilus T6SS core components, related to Figure 1A

Table S3. List of label-free quantified proteins in mono-species biofilm, related to Figures 2E and 2F

Table S4. List of GO terms from all A. aphrophilus proteins documented by UniProt, related to Figures 2E and 2F

Table S5. Summary of all GO terms from mono-species biofilms, related to Figures 2E and 2F

Table S6. The numerical composition of the individual bacterial species in the biofilms, related to Figures 3B and 3C

Table S7. List of label-free quantified proteins in multi-species biofilm, related to Figure 4A

Table S8. List of GO terms from all bacterial proteins documented by UniProt, related to Figures 4B and 4C

Table S9. Summary of all GO terms from multi-species biofilms, related to Figures 4B and 4C

Table S10. Significant correlation between T6SS core proteins with other proteins, related to Figures 4F and 4G

Table S11. List of UPID and NCBI taxon identifiers for each database, related to methods details in STAR Methods
